# Pituitary society guidance: pituitary disease management and patient care recommendations during the COVID-19 pandemic—an international perspective

**DOI:** 10.1007/s11102-020-01059-7

**Published:** 2020-06-18

**Authors:** Maria Fleseriu, Michael Buchfelder, Justin S. Cetas, Pouneh K. Fazeli, Susana M. Mallea-Gil, Mark Gurnell, Ann McCormack, Maria M. Pineyro, Luis V. Syro, Nicholas A. Tritos, Hani J. Marcus

**Affiliations:** 1 Pituitary Center, Oregon Health & Science University, Mail Code CH8N, 3303 South Bond Ave, Portland, OR 97239 USA; 2 Department of Neurosurgery, University Hospital Erlangen, Erlangen, Germany; 3 Veterans Affairs Medical Center, Portland, OR USA; 4 Neuroendocrinology Unit, Division of Endocrinology and Metabolism, University of Pittsburgh, Pittsburgh, PA USA; 5 Division de Endocrinología, Hospital Militar Central, Buenos Aires, Argentina; 6 Addenbrooke’s Hospital, University of Cambridge and NIHR Cambridge Biomedical Research Centre, Wellcome Trust-MRC Institute of Metabolic Science, Cambridge, UK; 7 Department of Endocrinology, St Vincent’s Hospital, Sydney, Australia; 8 Garvan Institute of Medical Research, Sydney, NSW Australia; 9 Facultad de Medicina, Hospital de Clínicas, Clínica de Endocrinología Y Metabolismo, Universidad de La República, Montevideo, Uruguay; 10 Department of Neurosurgery, Hospital Pablo Tobon Uribe and Clinica Medellin – Grupo Quirónsalud, Medellin, Colombia; 11 Neuroendocrine Unit and Neuroendocrine and Pituitary Tumor Clinical Center, Massachusetts General Hospital and Harvard Medical School, Boston, MA USA; 12 National Hospital for Neurology and Neurosurgery, London, UK

**Keywords:** SARS-CoV-2, COVID-19, Pituitary disease and surgery, Cushing’s disease, Acromegaly, Prolactinomas

## Abstract

Severe acute respiratory syndrome coronavirus 2 (SARS-CoV-2), the viral strain that has caused the coronavirus disease 2019 (COVID-19) pandemic, has presented healthcare systems around the world with an unprecedented challenge. In locations with significant rates of viral transmission, social distancing measures and enforced ‘lockdowns’ are the new ‘norm’ as governments try to prevent healthcare services from being overwhelmed. However, with these measures have come important challenges for the delivery of existing services for other diseases and conditions. The clinical care of patients with pituitary disorders typically involves a multidisciplinary team, working in concert to deliver timely, often complex, disease investigation and management, including pituitary surgery. COVID-19 has brought about major disruption to such services, limiting access to care and opportunities for testing (both laboratory and radiological), and dramatically reducing the ability to safely undertake transsphenoidal surgery. In the absence of clinical trials to guide management of patients with pituitary disease during the COVID-19 pandemic, herein the Professional Education Committee of the Pituitary Society proposes guidance for continued safe management and care of this population.

## Introduction

In many centers worldwide, the evaluation and treatment of pituitary disorders has already been substantially impacted by severe acute respiratory syndrome coronavirus 2 (SARS-CoV-2), the viral strain that has caused the coronavirus disease 2019 (COVID-19) pandemic. With reduced access to routine clinical services, patients with suspected or confirmed pituitary disease face the prospect of delays in diagnosis and implementation of effective treatment plans. Furthermore, patients undergoing surgery may be at increased risk from COVID-19, whilst the risk of infection to healthcare providers during pituitary surgery is of particular concern.

Herein, we discuss several clinical scenarios where clinical care can be adjusted temporarily without compromising patient outcomes. For this expert guidance, The Pituitary Society Professional Education Committee, which includes neuroendocrinologists and neurosurgeons from four continents, held an online video conference call with subsequent discussions conducted through email communications. The suggestions are not evidence-based due to the novelty and timing of the pandemic; furthermore, re-evaluation every few months in light of emerging data, is recommended. The approach will also likely vary from country to country depending on the risk of viral infection, local rules for “lockdown”, and the capabilities of individual health care systems.

### Pituitary surgery challenges during the COVID-19 pandemic

The significant challenges to pituitary surgery presented by COVID-19 can be considered in terms of the phase of the pandemic, the patient, the surgeon, and the healthcare institution (Table 1).

**Table 1:** Pituitary surgery challenges and recommendations during COVID-19 pandemic

Factor	Challenges	Recommendations
COVID-19	High prevalence of cases in the community during pandemic and risk of additional waves in the post-peak phase	Screening for cough, fever, and other symptoms and, if suspected, swab for testing *Consider* Isolation up to two weeks before surgery Paired swabs for testing and/or serological tests Chest X-ray and/or chest CT*
Patient	High risk of older patients with comorbid conditions contracting COVID-19; consider natural history of pituitary disease	Emergency surgery if pituitary apoplexy, acute severe visual loss or other evidence of significant mass effect, or if there is concern regarding malignant pathology *Consider* Surgery for patients with less acute, but progressive visual loss, functioning tumors with aggressive clinical features, and those with an unclear diagnosis
Surgeon	Risk of surgeon contracting COVID-19 from patient	In a patient with COVID-19 that requires emergent surgery that cannot be deferred, alternative transcranial approaches may be considered, drilling avoided, and full PPE is mandated *Consider* Full PPE in all cases
Institution	Diversion of resources to (non-pituitary) patients with COVID-19	Maintain flexibility for second wave

*PPE* personal protective equipment

*Depending on local guidance, chest CT is mandatory in some centers

The World Health Organization (WHO) recognizes several phases of a *pandemic* wave [1]. When the pandemic is in progress (WHO pandemic phase descriptions; Phase 6) [2] there is a high prevalence of active cases. In the immediate post-peak period, the pandemic activity appears to wane, but active cases remain, and additional waves may follow. Previous pandemics have had many such waves, each separated by several months (www.cdc.gov). The corollary is that there will remain a significant possibility of patients and surgeons contracting COVID-19 until a vaccine is developed or herd immunity is achieved by other means.

The *patient* requiring pituitary surgery may be especially vulnerable to COVID-19 due to age and/or comorbidities. This is particularly true of patients with functioning pituitary adenomas such as those with Cushing’s disease (CD), where cortisol excess results in immunosuppression, hypercoagulability, diabetes mellitus and hypertension, and acromegaly which is also frequently complicated by diabetes mellitus and hypertension. Moreover, the risk for patients undergoing surgery that develop COVID-19 in the perioperative period appears to be very high. In a retrospective analysis of 34 patients who underwent elective—non pituitary—surgeries during the incubation period of COVID-19, 15 (44.1%) patients required admission to the intensive care unit, and 7 (20.5%) died [3]. Although this study included cases of variable technical difficulty, complexity and risk—from excision of breast lump to total hip replacement—we would suggest that patients undergoing pituitary surgery that develop COVID-19 are likely to be at similar or greater risk. These risks must be balanced carefully against the natural history of pituitary disease and, in particular, whether undue delay may result in irreversible morbidity such as visual loss in patients with pituitary apoplexy.

The *surgeon* remains in direct contact with the patient throughout their operation and is therefore at risk of contracting COVID-19 if the patient has an active infection. Iorio-Morin et al. [4] suggest that surgeons performing transsphenoidal pituitary surgery (TSS) may be at the greatest risk, because such surgery is performed under general anesthesia, requiring intubation and extubation, exposes the colonized nasal mucosa, and usually involves sphenoid drilling, which can result in aerosolization of contaminated tissues.

The *healthcare institution* will invariably divert resources from elective services to support the care of patients with COVID-19, with a knock-on effect on the capacity to manage patients with pituitary disease (Table 1). Bernstein et al. [5] suggest that surgery is particularly affected in such reorganization, because of both the need for redeployment of anesthesiologists able to manage patient airways, and availability of protective physical resources such as masks, gowns, and gloves (personal protective equipment; PPE). Furthermore, in areas with high number of infections, several operating rooms (OR)s were converted into intensive care units (ICU) to treat patients with COVID-19, thus limiting patients’ access to elective surgery even more.

### Recommendations for pituitary surgery

When the viral risk is decreasing in a specific geographic area, we would advocate a stepwise, but flexible normalization of activity, addressing each of the aforementioned factors.

Burke et al. [6] proposed a staged volume limiting approach to scheduling surgical cases depending on the number of community cases and inpatients with COVID-19, and staffing shortages. In extreme cases, where significant assistance is required from outside institutions, only emergent cases can proceed.

Until further data become available, all patients undergoing pituitary surgery should undergo screening for COVID-19, until a vaccine is developed or herd immunity is achieved by other means. At the least, we recommend screening patients for cough, fever, or other recognized symptoms of infection with SARS-CoV-2, and taking swab samples for testing if there is any clinical suspicion. Depending on the level of COVID-19 activity in the community, and available resources, a more exhaustive strategy may be appropriate, including isolation of patients for up to 2 weeks before surgery, paired swabs and/or serological tests for all patients irrespective of symptoms, and routine chest X-ray or chest computed tomography (CT), depending on local guidance. In patients with COVID-19 in whom surgery is indicated, in general we recommend delaying surgery if possible, ideally until patients no longer have symptoms and have a negative swab test result.

The nature of the patient’s pituitary disease is an important consideration, and we propose stratifying cases as emergent, urgent, or elective. We recommend that patients continue to be operated on in an emergent fashion if they present with pituitary apoplexy, acute severe visual loss, or other significant mass effect, or if there is concern regarding malignant pathology. Selected patients with slowly progressive visual loss, functioning tumors with aggressive clinical features, and those with an unclear diagnosis, may also benefit from urgent (but not emergent) surgery, with decisions made on a case-by-case basis. Patients with incidental and asymptomatic tumors, known nonfunctioning adenomas [7] or functioning tumors, which are well controlled with medical therapy, can be scheduled as elective cases.

In most cases, TSS remains the safest, most effective, and most efficient approach to pituitary tumors. In a series of 9 consecutive patients without COVID-19 undergoing pituitary and skull base surgery during the pandemic, Kolias et al. [8] reported that none of the patients or staff contracted COVID-19 following adoption of a standardized risk-mitigation strategy. In the rare instances where a patient with COVID-19 requires emergent surgery that cannot be deferred, alternative transcranial approaches may be considered (avoiding nasal mucosa). To replace high-speed drilling, the use of non-powered tools such as rongeurs and chisels has been recommended. If this is not possible large suction tubes can be used to aspirate as much particulate matter as possible [9]. In such cases, the availability and use of PPE, and in particular filtering facepiece (FFP3) respirators, is mandated. Depending on the level of COVID-19 activity in the community, and the availability and effectiveness of testing, PPE may be appropriate in all cases.

At an institutional level, there must remain flexibility in anticipation of further waves of COVID-19. This necessitates a reduction in capacity, particularly in available ICU beds, that must be recognized when scheduling challenging surgical cases. In the long term, resumption of full elective workloads depends on wider national and international factors, including widespread testing, and widespread immunity through vaccination or other means.

### Pituitary diseases diagnosis and management

#### Acromegaly

Acromegaly, a condition that arises from growth hormone (GH) excess, generally occurs as a result of autonomous GH secretion from a somatotroph pituitary adenoma [10, 11], is associated with substantial morbidity and excess mortality, which can be mitigated by prompt and adequate treatment [12]. Diagnosis is often delayed because of the low prevalence of the disease, the frequently non-specific nature of presenting symptoms, and the typically subtle progression of clinical features [10, 11]. During the COVID-19 pandemic many outpatient clinics have closed or limited work hours. Patients are often reluctant to seek care out of fear of possible exposure to the coronavirus. Therefore, even longer diagnostic delays are anticipated. In addition, patients who present with vision loss and larger tumors encroaching upon the optic apparatus are at risk for experiencing persistent visual compromise unless the optic chiasm and nerves are promptly decompressed.

To improve patient access to care and minimize potentially deleterious delays in diagnosis and treatment, clinicians may conduct virtual visits (VV) using secure, internet-based electronic medical record platforms. A detailed history can be obtained and a limited physical examination is possible, including inspection of the face, skin and extremities.

#### Diagnosis

Establishing the diagnosis of acromegaly requires testing of serum insulin-like growth factor-I (IGF-I) levels [11] (Box 1). Access to accurate IGF-I assays is critical in light of the substantial analytical and post-analytical problems that have plagued several IGF-I immunoassays. While the oral glucose tolerance test (OGTT) is considered the diagnostic “gold standard”, this test is not essential in many patients, including those with a clear-cut clinical picture and an unequivocally elevated serum IGF-I level. Deferring the lengthy (2-h) OGTT may minimize the risk of potential exposure to infectious agents.

Given the over-representation of macroadenomas in patients with acromegaly, pituitary imaging is indicated, preferably by a pituitary-specific magnetic resonance imaging (MRI) protocol, although CT may be performed to rule out a large tumor if MRI is not feasible. Obtaining imaging at satellite sites detached from major hospitals may also decrease the risk of infection exposure.

#### Management

Transsphenoidal pituitary surgery remains the treatment of choice for most patients with acromegaly [10, 11], and patients with visual compromise as a result of a pituitary adenoma compressing the optic apparatus should still undergo pituitary surgery promptly. Other patients could be treated medically until the pandemic subsides. Medical treatment options are somatostatin receptor ligands (SRLs), octreotide long-acting release (LAR), lanreotide depot and pasireotide LAR, pegvisomant and cabergoline (used off-label) [13]. Medical therapies can be effective in providing symptomatic relief, control GH excess or action, and potentially reduce tumor size (except pegvisomant, which does not have direct antiproliferative effects). Preoperative medical therapy has been reported to improve surgical outcomes in some, but not all studies. Pasireotide, which potentially can induce QTc prolongation, should be used with caution in patients who are taking, either as prophylaxis or treatment, medications for COVID-19 (azithromycin, hydroxychloroquine), which can also have an effect on QTc interval. Furthermore, as hyperglycemia is very frequent in patients treated with pasireotide and needs close monitoring at start of the treatment, this treatment should be reserved for truly resistant cases, with large tumors and who cannot have surgery yet. Notably, lanreotide depot, cabergoline or pegvisomant can be administered by the patient or a family member and therefore an in-person visit to a clinic is not required. If SRLs that require health care professional administration are required, raising the dose may allow the interval between injections to be extended beyond 4 weeks while maintaining disease control. Virtual visits can be implemented to monitor the patient’s course and response to medical therapy during the pandemic. Careful management of comorbidities associated with acromegaly remains an essential part of patient care [14, 15].

#### Prolactinomas

Hyperprolactinemia may be physiological in origin or arise because of an underlying pathophysiologic cause, medication use or laboratory artifact. Therefore, an initial evaluation for hyperprolactinemia should include a comprehensive medication history, a thorough evaluation for secondary causes, including primary hypothyroidism, and a careful assessment for clinical features of hyperprolactinemia, including hypogonadism and galactorrhea. Unless a secondary cause of hyperprolactinemia can be established definitively, further investigation is indicated to evaluate the etiology of hyperprolactinemia.

#### Diagnosis

The diagnosis of a lactotroph adenoma can be inferred in most patients based on the presence of a pituitary adenoma and an elevated prolactin level, which is typically proportionate in magnitude to adenoma size. Pituitary imaging (MRI or CT) is therefore a key step in the investigation of hyperprolactinemia. Evaluation for hypopituitarism is also necessary.

#### Management

Although observation and routine follow-up with serial prolactin levels and imaging is acceptable for patients who are asymptomatic and who have a microadenoma, most patients diagnosed with a prolactinoma will require treatment. Dopamine-agonists (DA) can normalize prolactin levels and lead to reduction in size of the lactotroph adenoma [16]. In patients who have a microadenoma and who are not seeking fertility, hormone-replacement therapy may also be appropriate if serum prolactin is routinely followed and imaging performed as necessary.

Medical therapy can be managed effectively and efficiently via VVs coupled with laboratory/imaging studies as needed. However, in all patients in whom a DA will be initiated, it is critical that a comprehensive psychiatric history is obtained prior to commencing treatment. Patients may not readily volunteer their psychiatric history and may not appreciate the relevance of such information. For example, until specifically questioned about their psychiatric history, the patient described in the illustrative case (Box 2) did not report a history of severe depression, suicide attempt and prolonged psychiatric hospitalization 8 months prior to presentation with hyperprolactinemia. At the time of the visit, he was not taking any psychiatric medications and was not under the care of a mental health team. Given this patient’s significant psychiatric history, lack of ongoing psychiatric care, and the well-recognized adverse effects of DA therapy, including increased impulsivity, depression and psychosis [17], a DA was not initiated. Counseling on potential DA side-effects is crucial, as they may also present in individuals with no prior psychiatric history [17]. Furthermore, during the COVID-19 pandemic when there is reduced access to routine medical and mental health care, patients who develop symptoms of severe depression may not have ready access to mental health services, or may not seek care. Therefore, it is particularly important to make patients aware of these potential side effects and the critical importance of reporting them.

In the small number of patients for whom medical therapy is not possible and where surveillance is not appropriate (e.g., macroprolactinoma with visual loss) the risks and benefits of surgical intervention will need to be carefully weighed.

#### Cushing’s disease

Left untreated, CD has significant morbidity and mortality, and delays in diagnosis (from a few months to even years) are common. Clinical presentation is also very variable with some patients having subtle symptoms while others present with more striking/classical features. Severe hypercortisolemia induces immunosuppression, which may place patients with untreated CD at particular risk from COVID-19.

### New patients referred for endocrinology evaluation with clinical suspicion of Cushing’s

#### Diagnosis

Screening for, and confirmation of Cushing’s syndrome (CS) and, furthermore, localization for CD is laborious and requires serial visits and testing procedures [18, 19]. If initial laboratory abnormalities are consistent with hypercortisolemia, a VV should allow for an estimate of the severity of clinical presentation and facilitate planning for further testing and treatment. Careful questioning for potential causes of exogenous CS (including, but not limited to, history of high-dose oral corticosteroids, intraarticular injections or topical preparations) is an important first step. Subsequently, establishing the likelihood and pretest probability of CS is more important than ever now, when testing may be delayed. While presentation varies significantly between patients, some features, although not all highly sensitive, are more specific, e.g. easy bruising, facial plethora, large wide > 1 cm violaceous striae, proximal weakness and hypokalemia. Diagnosis of CS is often challenging even under normal circumstances, however, a diagnosis by VV is more nuanced and difficult. Conversely, if a patient has a high likelihood of CS, we recommend limited laboratory evaluation (urinary free cortisol (UFC), adrenocorticotropic hormone (ACTH), liver panel, basic metabolic panel), preferably at a smaller local laboratory rather than a Pituitary Center, to reduce viral risk exposure. Salivary cortisol samples could represent a hazard for laboratory staff and they are prohibited in some countries [18, 19]. In the US, laboratories have continued to process salivary cortisol samples and salivary cortisol has higher sensitivity compared with UFC and has the convenience of mailing multiple specimens at a time, without travel [18, 19]. Though usually we strongly recommend sequential laboratory testing under normal circumstances, limiting trips to a laboratory is preferred during COVID-19.

If preliminary assessment confirms ACTH-dependent CS [18, 19] and no visual symptoms are reported, imaging may be delayed. However, in the presence of any visual symptoms, and recognizing the challenges of undertaking a formal visual field assessment, proceeding directly with MRI or CT (shorter exam time and easier machine access) imaging, will allow confirmation or exclusion of a large pituitary adenoma compressing the optic chiasm. If the latter is confirmed, the patient will need to be evaluated by a neurosurgeon. In contrast, a small pituitary adenoma may not be visible on CT, but in such cases MRI may be deferred for a few months until COVID-19 restrictions limiting access to care are lifted.

Another VV will help to decide, in conjunction with patient’s preference, the best next step, which in cases of more severe clinical Cushing’s, and in the absence of a large pituitary adenoma, would be medical therapy. The magnitude of 24 h-UFC elevation could also represent a criterion for primary therapy, since higher values have been associated with increased risk of infection.

In parallel, it is also important to address comorbidities including diabetes mellitus, hypertension and hyperlipidemia. In light of the increased risk of venous thromboembolism, in discussion with primary care providers, plans for regular mobilization/exercise as permitted (including at home when orders to stay in are in place) and/or prophylactic low weight molecular heparin should be considered.

#### Management

First line medical therapy options vary, depending on country availability, regulatory approval and patient comorbidities. Ideally, an oral medication, which is easier to administer is preferred; options include ketoconazole, osilodrostat or metyrapone [20, 21]. Cabergoline therapy, which has lesser efficacy [20, 21] compared with adrenal steroidogenesis inhibitors, can be also attempted in very mild cases. The initial laboratory profile should be reviewed to exclude significant abnormalities of renal and/or liver function prior to commencing treatment. Starting doses of all medications should be the lowest possible to avoid adrenal insufficiency (AI) and up titration should be slow, with VVs weekly if possible. All patients with CS on any type of medical therapy should have prescribed glucocorticoids (GC) both in oral and injectable forms available at home and information regarding AI should be provided during a VV when starting therapy for CS. Down titration of other medications for diabetes and hypertension may also be needed over time. Pasireotide (both subcutaneous and LAR preparations) would be a second line option, reflecting higher risk of significant hyperglycemia that would require treatment [22].

If the clinical features of CS are mild and longstanding, with no acute deterioration, another possibility is to aggressively treat the associated comorbidities for a few months; depending on local circumstances, this may actually be less risky for the patient by avoiding the risk of AI/crisis and the need for an emergency department (ED) visit and/or admission.

### For patients with Cushing’s disease with endocrinology chronic care

#### Patients in remission after surgery with adrenal insufficiency on glucocorticoid replacement

These patients are likely to remain at slightly higher risk of COVID-19 infection due to immunosuppression from previous hypercortisolemia. Furthermore, GC doses should be adjusted to prevent adrenal crisis and visits to an ED. Lower GC daily doses (10–15 mg hydrocortisone/day) are now frequently used for replacement and virtual and/or phone visits are encouraged to evaluate an appropriate regimen and sufficient supplies of medication and injectable GC (at home) should be prescribed. Patients with potential symptoms of under replacement may require an increase in daily dose, while balancing any risk of GC over replacement and possible consequent immunosuppression.

#### Patients in non-remission treated with medical therapy (dependent on country availability)

Doses may need to be adjusted to reduce the risk of AI/crisis and reduce the need for serial laboratory work. Monthly or bimonthly VVs are appropriate for clinical evaluation and up titration should be slower than usual. Patients with CD on medical therapy need to have at home prescriptions for oral and injectable GC and instruction on AI surveillance. Patients should also be advised, that if they develop a fever, to stop Cushing’s medication for few days; if they develop AI symptoms, GC administration will be required. In some countries, block and replace regimens are also employed to avoid risk of AI. Of note, for mifepristone, a glucocorticoid receptor (GR) antagonist, patients will require much higher doses of GC to reverse the blockade (1 mg of dexamethasone approximately per 400 mg of mifepristone) and for several days, as drug metabolites also have GR antagonist effects.

Furthermore, for all patients who have made dose changes or discontinued medications for Cushing’s, it is essential to follow very closely and consider adjustments in the doses of concomitant medications, especially insulin, other antidiabetic and antihypertensive medications, and potassium supplements.

If patients have history of radiotherapy and are still on medications for CD, a VV every few months should be performed to determine if anti-Cushing’s treatment can be slowly down-titrated (to avoid AI). A morning serum cortisol would be ideal to rule out AI off medications, however, if laboratory testing cannot be undertaken safely, clinical evaluation by serial VVs can be helpful. While head-to-head data will never be available, in COVID-19 hotspots, given the higher risk of infection with laboratory testing or face to face visits, mild hypercortisolemia might be “better” than adrenal crisis, especially in the short term!

Patients with CD have increased rates of depression, anxiety and can have decreased quality of life (QoL) even when in long-term remission, thus in the challenging circumstances of the current pandemic it is it even more important to focus on psychological evaluation during virtual endocrinology visits, with referral to virtual counseling as needed.

Box 1 Acromegaly—illustrative case Clinical case: A 52-year-old man presented with several months’ history of frequent, frontal headaches that persisted despite trials of preventive therapies, including amitriptyline and topiramate. He had been diagnosed with hypertension and sleep apnea 2 years prior but had no known diabetes mellitus. He was taking lisinopril and topiramate. His neurologist obtained a brain MRI examination, which demonstrated a sellar mass consistent with an adenoma, measuring 1.2 cm in greatest diameter; the tumor was not impinging on the optic chiasm. His primary care physician noticed coarsening of patient’s facial features in comparison with older photographs and referred him to an endocrinologist.

Question: *How should this patient be evaluated and managed in the setting of an ongoing pandemic?*

Case follow-up: The patient had an elevated serum IGF-I level, which was 2.5 times above the upper end of the reference range (xULN). There was no laboratory evidence of other pituitary hormone deficits. Treatment with lanreotide depot 120 mg every 4 weeks, which is easier to self -administer or be administered by a family member, led to substantial improvement in headaches and IGF-I normalization within 12 weeks. He was referred to consult with an expert neurosurgeon and discuss undergoing TSS electively, when this becomes feasible.

Box 2 Prolactinoma—illustrative case Clinical case: A 38-year-old man presented with a two-year history of fatigue and low libido. An 8 AM testosterone level was low at 225 ng/dL (reference range; 250–1100 ng/dL) and a prolactin level was subsequently found to be 700 ng/mL (reference range; < 20 ng/mL). Pituitary MRI demonstrated a 1.2 cm pituitary adenoma with suprasellar extension, abutting, but not compressing, the optic chiasm. After further discussion with the patient, a long-standing history of severe psychiatric disease, not treated at this time, is revealed.

Question: *How should this patient be managed in the setting of an ongoing pandemic?*

Case follow-up: Given the size of the lesion (> 1 cm), laboratory studies for hypopituitarism were recommended and other than low testosterone, pituitary function was normal. He was referred for neurosurgical evaluation, the therapeutic option of choice if DAs are contraindicated or not effective. Unless visual field abnormalities or deficits become apparent in the interim, given the potential surgery risk, this patient could also be closely monitored while safely deferring surgery by repeating a prolactin level and pituitary MRI in 3 months.

## TSH-secreting pituitary adenomas

Thyroid stimulating hormone (TSH)-secreting pituitary adenomas are a rare but important cause of hyperthyroidism (estimated prevalence 2.8 per million population) [23, 24]. Clinical features resemble those observed in primary hyperthyroidism but without disease-specific (e.g. Graves ophthalmopathy) manifestations [24, 25]. Visual disturbance may complicate macroadenomas with suprasellar extension, whilst hormonal co-secretion (e.g. GH, prolactin) and/or hypopituitarism may also be evident [24, 25].

### Diagnosis

A major challenge in the diagnostic pathway is the exclusion of other common (e.g. laboratory assay interference, confounding medications, intercurrent illness) and rarer (e.g. *THRB* resistance to thyroid hormone; *THRB* RTH) causes of hyperthyroxinemia with non-suppressed TSH [25, 26]. This distinction remains important; avoid inappropriate investigation and treatment, and begin with a careful reappraisal of the clinical history and medications. Laboratory assay interference should be actively sought [25–27] and laboratories should be aware of the specific concern (e.g. serum dilution studies or polyethylene glycol (PEG) precipitation to exclude a spurious TSH result in the context of primary thyroid disease) [26, 27].

In a patient with visual disturbance suggestive of optic chiasmal compression and genuine hyperthyroxinemia with non-suppressed TSH (± evidence of other pituitary dysfunction), pituitary imaging should be performed (either MRI or, depending on local circumstances, dedicated pituitary CT) to confirm the presence of a macroadenoma with suprasellar extension.

In a patient without evidence of visual pathway compromise or associated hypopituitarism, a local decision will need to be made whether to attempt further investigation or defer until the COVID-19 pandemic subsides (in which case empiric treatment should be considered—see below). When further investigation is considered appropriate, the reader is directed to other publications that consider in detail how to discriminate between a TSH-secreting adenoma and *THRB* RTH [25–27].

### Management

As with other anterior pituitary tumor subtypes, the goals of treatment are control of hormone excess, amelioration of compressive symptoms and correction of hypopituitarism [24, 25, 28]. For patients with a macroadenoma and visual compromise, early surgical intervention remains the treatment of choice, once appropriate medical therapy has been instituted to mitigate the risks of anesthesia in a patient with uncontrolled thyrotoxicosis.

Medical treatment options depend on the clinical context. When a diagnosis is clear, first generation SRLs, e.g. octreotide LAR, lanreotide depot (which may constitute off-label use in some countries) are effective in rapidly correcting hyperthyroxinemia in the majority of patients (typically within few weeks). Such medications may be deployed in preparation for surgery or as primary medical therapy until surgery becomes feasible (NB; tumor shrinkage is variable with SRL therapy, and surgery therefore remains the best treatment to rescue/preserve vision). As with acromegaly, DA therapy (e.g. cabergoline used off-label) may be effective in some cases (especially when there is genuine prolactin co-secretion).

For those patients in whom diagnosis is suspected, but not confirmed, a SRL trial may prove both diagnostic and therapeutic. Alternatively, and especially in those with palpitations/arrhythmias or those deemed at risk of cardiovascular complications, non-selective beta-blockers are a reasonable option (with non-dihydropyridine calcium channel blockers reserved for those in whom beta-blockade is contraindicated). Appropriate consideration must also be given to anticoagulation in those subjects with atrial fibrillation, who are likely to be at the same excess risk of thromboembolic events as patients with primary thyroid disease.

A role for conventional anti-thyroid drug therapy in the management of TSH-secreting pituitary adenomas remains controversial, as tumor expansion is at least theoretically possible, although short-term usage, especially in those with a microadenoma, may be appropriate in some cases (e.g. when there is resistance to SRL therapy).

## Hypopituitarism

Clinicians need to remain vigilant in managing patients with hypopituitarism during the COVID-19 pandemic. There is an increased background mortality rate among patients with hypopituitarism, particularly due to cerebrovascular and cardiovascular disease, which is an important risk factor for COVID-19 disease severity [29, 30]. There is also a careful balance in managing the threat of AI at this time with prolonged GC over-replacement and consequent immunosuppression. Patients with diabetes insipidus (DI) present a particular challenge in fluid balance during significant illness, with hypernatremia linked to increased mortality in those admitted to intensive care units [31].

### Diagnosis

In a patient with a newly detected pituitary lesion or suspected hypopituitarism, an early morning fasted pituitary profile should be arranged promptly with a local laboratory and results can be discussed at a VV. If DI is strongly suspected based on clinical history a non-fasted morning blood sample for serum sodium and osmolality should be collected first. Patients with complete DI often present with hypernatremia in this setting, avoiding the need for a protracted water deprivation test. A cortisol level < 3 µg/dL indicates AI while a cortisol level > 15 µg/dL reliably excludes AI [29]. Dynamic testing is ordinarily performed to determine adequacy of the hypothalamic–pituitary–adrenal axis with intermediate results, however there may be a significant delay in obtaining a short cosyntropin test, or insulin tolerance test, at this time with health care systems limiting direct exposure of patients to health care personnel. A low free T4 in concert with a low/normal TSH is suggestive of central hypothyroidism. In non-obese males with a pituitary lesion, low serum testosterone and low/normal gonadotrophins likely indicate central hypogonadism. Low estradiol with low/normal gonadotrophins in a pre-menopausal woman with amenorrhea (excluding other causes such as hypothalamic amenorrhea and hyperprolactinemia) or in a post-menopausal female also suggest central hypogonadism. Growth hormone deficiency is highly likely (even with a normal IGF-1 level) to co-exist in a patient with deficiencies of 3–4 pituitary axes although dynamic testing to confirm GH deficiency should be deferred for 6–12 months until specialized endocrine testing units are once again conducting these non-urgent procedures.

### Management

The safest option is to commence hydrocortisone replacement with 15–25 mg/day in divided doses. While over replacement is always important to avoid, in areas with high risk of viral exposure, it would be preferable to err on the side of starting on the higher end of this dose range to ensure no under replacement. Given the risk of an adrenal crisis with COVID-19 (or any other illness), all patients on GC should be reminded about sick day management [32]. This includes prompt doubling of their regular GC dose at the first sign of sickness, ensuring access to injectable GC and use of medical alert jewelry. Where medication supply may become an issue, it would be reasonable to make sure patients have 2–3 months reserves of their GC therapy and there may be a need to switch between GC preparations (for example hydrocortisone to cortisone acetate or prednisone depending on country approvals) if availability becomes limited. Adequate GC management can be assessed clinically by VV.

Once GC is started, it would be safe to commence thyroid replacement if required. A reasonable starting dose, in the absence of coexistent significant cardiac disease, would be approximately 50–75 µg levothyroxine daily, but dose is usually weight-based. Dose adjustment, and clinical reassessment, can take place via VV, aiming for a free T4 level in the mid-upper normal range [29]. Ideally, free T4 levels would be assessed 6–8 weeks later, however deferring for 3–4 months would be reasonable.

Testosterone or estrogen replacement could also reasonably be deferred for 6 months, although if there are significant symptoms persisting after GC and thyroid replacement, commencement of transdermal preparations would be advised over injectable testosterone to again limit the need for patients to visit primary care physicians. GH replacement is usually deferred in a patient with an untreated sellar mass. In patients already established on gonadal or GH replacement, clinical assessment to avoid side effects would be adequate, although it may be appropriate to ensure adequate testosterone levels in men and maintenance of IGF-1 below the age-adjusted upper normal reference level whenever essential biochemical testing is performed [29].

In patients with DI without concomitant illness the main risk is iatrogenic hyponatremia [29]. If access to regular electrolyte monitoring is difficult it would be prudent to advise patients to withhold a weekly dose of desmopressin to allow for an aquaresis and favor under-dosing during daytime, with an aim to relieve symptoms of polyuria particularly overnight. In those with mild or partial DI, treatment with desmopressin may not be required as long as patients are instructed to drink to thirst and have access to fluids. In hospitalized patients, hypernatremia is more likely to occur, particularly in those with adipsic DI, as patients may have reduced fluid intake, fever may increase water losses and oral desmopressin dosing may be more difficult. Regular electrolyte monitoring is required, with requirement for parenteral desmopressin and intravenous fluids in cases of severe illness.

## Conclusion

Taking care of patients with pituitary disorders typically involves a multidisciplinary approach in order to achieve timely, often complex, diagnostic and treatment plans, including pituitary surgery. The COVID-19 pandemic has significantly limited, in many regions, patients’ access to testing (laboratory and imaging), and severely decreased the capability to safely perform transsphenoidal surgery. Clinicians have expanded VV and new criteria and processes are being applied to stratify patients’ needs both for medical therapy and pituitary surgery.

The guidance provided here is based on rapid expert consensus of the Pituitary Society Professional Education Committee, however, due to the emerging nature of the COVID-19 crisis, information can change very rapidly. Providers and patients alike need to always consider individual patients’ conditions, and local country and region particularities, both for viral load and healthcare availabilities when devising individualized patient management plans.
